# P-1091. Codon Bias Variation within Staphylococcus aureus

**DOI:** 10.1093/ofid/ofaf695.1286

**Published:** 2026-01-11

**Authors:** Kathleen O’Connor, Benjamin D Mallinger, Nicholas Bedard, Robin Patel

**Affiliations:** Mayo Clinic, Rochester, Minnesota; Mayo Clinic, Rochester, Minnesota; Mayo Clinic, Rochester, Minnesota; Mayo Clinic, Rochester, Minnesota

## Abstract

**Background:**

*Staphylococcus aureus* is a challenging pathogen which causes a multiplicity of human diseases around the globe, acquired in the community and healthcare settings alike. Treatment of *S. aureus* infection can be challenging and is further compromised by antimicrobial resistance. Understanding of its pathogenesis, transmission pathways and the dynamics of its genome evolution and associated adaptations is incomplete. One strategy to assess genome evolution is to assess codon bias. Historically, studies have focused on the consequences of coding changes; assessing silent mutations in the form of synonymous codon usage bias represents a novel approach to studying genetic adaptation.

**Figure 1.**
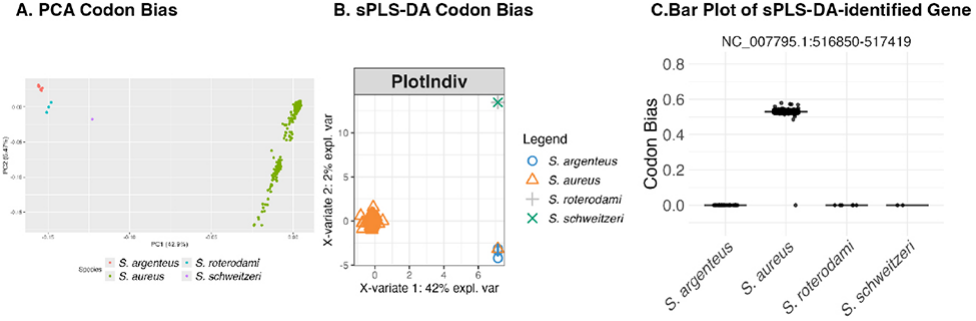
Principal component analysis (PCA) (A) and sPLS-DA (B) separate S. aureus from complex mates. sPLS-DA component 1 relies on a hypothetical protein, identified here by its NCTC 8325 genomic coordinates, as important for separating S. aureus from complex mates (C).

**Methods:**

Here, the codon bias of 2,872 genes was queried against all publicly available complete *S. aureus* plasmids and genomes. Using public BioSample data, genomes were sorted by disease state (e.g., bacteremia, pulmonary infection) and geographical collection site.

**Results:**

Isolates from Nairobi, Kenya were most different from other isolates, suggesting that geographical isolation may shape codon bias signature. In addition, codon bias of two MSCRAMM proteins implicated in human disease - SdrC and FnBPB - varied between isolation sources, suggesting a role for codon bias in *S. aureus* genetic adaptation to selective pressures of infection. Additionally, codon bias out-performed coding and nucleic acid mutational metrics in separating members of the *S. aureus* complex, *S. aureus*, *Staphylococcus argenteus*, *Staphylococcus schweitzeri*, and *Staphylococcus roterodami*; Fig 1, sparse partial least squares analysis (sPLS-DA) mean AUC codon: 0.89762, nucleic acid: 0.82266, codon bias: 0.9996 (relying on hypothetical protein NC_007795.1:516850-517419).

**Conclusion:**

This analysis suggests that codon bias in *S. aureus* is a source of genetic adaptation that can be used to separate strains by geographical isolation source, and separate *S. aureus sensu stricto* from other *S. aureus* complex members.

**Disclosures:**

Nicholas Bedard, MD, Stryker: Advisor/Consultant Robin Patel, MD, a patent on Bordetella pertussis/parapertussis PCR issued, a patent on a device/method for sonication, a patent on PET imaging of bacterial infection: a patent on Bordetella pertussis/parapertussis PCR issued, a patent on a device/method for sonication, a patent on PET imaging of bacterial infection|MicuRx Pharmaceuticals and bioMérieux: Grant/Research Support|PhAST, Day Zero Diagnostics, DEEPULL DIAGNOSTICS, S.L., Nostics, HealthTrackRx, bioMérieux and CARB-X: Advisor/Consultant|Up-to-Date and the Infectious Diseases Board Review Course: Honoraria

